# Combination of prostate cancer antigen 3 (PCA3), sarcosine, glypican-1 (GPC1), urokinase plasminogen activator receptor (uPAR), and thymidine kinase 1 (TK1), and T2WI and DWI radiomics model for distinguishing benign prostatic hyperplasia, prostate cancer, and prostatitis

**DOI:** 10.5937/jomb0-57639

**Published:** 2025-09-05

**Authors:** Fan Yang, Wei Guo, Siqin Sun, Yanan Huang

**Affiliations:** 1 Wuhan Third Hospital (Tongren Hospital of Wuhan University), Department of Radiology, Wuhan, Hubei 430070, China

**Keywords:** prostate cancer antigen 3 (PCA3), sarcosine, glypican-1 (GPC1), urokinase plasminogen activator receptor (uPAR), thymidine kinase 1 (TK1), prostatitis, benign prostatic hyperplasia, prostate cancer, biomarkers, radiomics, T2-WI, DWI, PI-RADS V, antigen karcinoma prostate 3 (PCA3), sarkozin, glipikan-1 (GPC1), receptor urokinaznog aktivatora plazminogena (uPAR), timidinska kinaza 1 (TK1), prostatitis, benigna hiperplazija prostate, karcinom prostate, biomarkeri, radiomiks, T2-WI, DWI, PI-RADS V2

## Abstract

**Background:**

To measure the diagnostic value of T2WI and DWI radiomics model, combined with advanced biomarkers, in distinguishing benign prostatic hyperplasia (BPH), prostate cancer (PCa) and prostatitis.

**Methods:**

A total of 90 patients with prostate diseases were selected from our hospital from January 2022 to January 2024. All patients underwent T2WI and DWI MRI examinations. Regions of interest (ROI) were delineated, and imaging features were extracted using radiomics analysis. In addition, novel biomarkers, including Prostate Cancer Antigen 3 (PCA3), Sarcosine, Glypican-1 (GPC1), Urokinase Plasminogen Activator Receptor (uPAR), and Thymidine Kinase 1 (TK1), were analysed for their diagnostic significance. Feature selection was performed using LASSO regression, and a random forest model was established for classification. The receiver operating characteristic (ROC) curve was used to evaluate the diagnostic performance of the T2WI and DWI radiomics model with and without biomarker integration.

**Results:**

Among the 90 patients with prostate diseases, 50 cases were PCa, 20 cases of prostatitis and 20 cases of BPH were detected by biopsy. The PI-RADS v2 score in the PCa group presented elevation relative to those in the BPH and prostatitis groups (P<0.01). The ADC values in the PCa group were reduced relative to those in the BPH and prostatitis groups (P<0.01). The integration of biomarkers with radiomics analysis led to improved diagnostic performance. The AUC value, sensitivity, and specificity of the T2WI and DWI radiomics model were higher relative to those of PI-RADS V2.

**Conclusions:**

The T2WI and DWI radiomics model, when combined with novel biomarkers, enhances the accuracy of distinguishing PCa, BPH, and prostatitis. This approach may provide an advanced diagnostic tool for personalised prostate disease management.

## Abstract (sr)

**Uvod:**

Cilj je bio da se ispita dijagnostička vrednost radiomiks modela zasnovanog na T2WI i DWI, u kombinaciji sa naprednim biomarkerima, u razlikovanju benigne hiperplazije prostate (BPH), karcinoma prostate (PCa) i prostatitisa.

**Metode:**

Ukupno 90 pacijenata sa oboljenjima prostate je izabrano u našoj bolnici u periodu od januara 2022. do januara 2024. Svi pacijenti su podvrgnuti T2WI i DWI MRI pregledima. Regije interesa (ROI) su bile definisane, a karakteristike slika ekstrahovane su korišćenjem radiomiks analize. Pored toga, analizirani su novi biomarkeri, uključujući Prostate Cancer Antigen 3 (PCA3), Sarkozin, Glipikan-1 (GPC1), receptor urokinaznog aktivatora plazminogena (uPAR) i timidinsku kinazu 1 (TK1), radi procene njihove dijagnostičke vrednosti. Selekcija karakteristika je izvršena korišćenjem LASSO regresije, a za klasifikaciju je primenjen model zasnovan na algoritmu "slučajne šume" (random forest). Korišćena je ROC kriva za procenu dijagnostičke efikasnosti radiomiks modela T2WI i DWI, sa i bez integracije biomarkera.

**Rezultati:**

Među 90 pacijenata sa oboljenjima prostate, biopsijom je utvrđeno 50 slučajeva PCa, 20 slučajeva prostatitisa i 20 slučajeva BPH. PI-RADS v2 skor je bio značajno viši u grupi sa PCa u poređenju sa grupama sa BPH i prostatitisom (P<0,01). ADC vrednosti su bile snižene u PCa grupi u poređenju sa BPH i prostatitis grupama (P<0,01). Integracija biomarkera sa radiomiks analizom dovela je do poboljšane dijagnostičke efikasnosti. Vrednosti AUC, senzitivnosti i specifičnosti radiomiks modela T2WI i DWI bile su više u poređenju sa PI-RADS V2.

**Zaključak:**

Radiomiks model T2WI i DWI, u kombinaciji sa novim biomarkerima, poboljšava tačnost u razlikovanju PCa, BPH i prostatitisa. Ovaj pristup može da bude napredni dijagnostički alat za personalizovano upravljanje bolestima prostate.

## Introduction

In recent years, it has been reported that the incidence of male prostate disease in China has been rising year by year, and there is a trend of younger men, which has gradually become a significant disease threatening the health of men in our country [1]. Prostate disease will affect the patient’s everyday life; severe cases will appear in frequency of urination, urination urgency, urine pain, urinary incontinence, kidney function damage, sexual dysfunction, and even uremia, affecting the patient’s life safety [2]. As an important tissue, prostate disease can be divided into benign and malignant lesions, among which benign prostatic hyperplasia (BPH) and prostate cancer (PCa) are the most common [3]. Because the prostate disease is entirely different in treatment and prognosis, especially since the early symptoms of PCa are not obvious, and the prognosis is poor in the middle and late stages, it is of great significance to identify the prostate disease early and receive appropriate treatment as soon as possible [4]. Simply observing clinical symptoms cannot accurately determine the type of lesions, and other examinations should be added for patients with prostate disease [5].

At present, there are in-depth clinical studies on prostate diseases, and CT has been popularised among various examination methods, which can observe prostate lesions at high resolution and master the function of the prostate [6]. However, the prostate structure is complex, and some small lesions are challenging to detect by CT. The radiation caused by this method is significant, which easily causes additional trauma to the prostate and other tissues and is not suitable for the diagnosis of prostate diseases [7]. Magnetic resonance has been popularised in recent years, which can observe the tissue structure of the prostate under high resolution and grasp the volume and location of the prostate lesions through high clarity, which can make accurate diagnoses for the specific types of prostate diseases [8]. Resonance examination techniques are varied, and T2WI sequence examination has been widely used in the past, which can observe the anatomical structure of prostate tissue and identify the lesion type according to the characteristics of image signals [9]. At present, DWI has received much attention. It can diffuse through water molecules during examinations and can be used to observe the abnormalities of prostate tissue, which is highly sensitive [10]. Some studies have pointed out that simple T2WI examination or simple DWI examination have disadvantages: the former has poor resolution, the latter is generally used more in the examination of cerebral infarction, and less in the examination of prostate and other organs, both of which may have the situation of missed diagnosis or misdiagnosis of prostate diseases, and the combined examination can complement each other and accurately identify prostate lesions [11].

Recent advances in biomarker research have introduced new molecular markers that, when combined with radiomics, can significantly improve the differentiation of BPH, PCa, and prostatitis. In addition to traditional markers like PSA, new biomarkers such as Prostate Cancer Antigen 3 (PCA3), Sarcosine, Glypican-1 (GPC1), Urokinase Plasminogen Activator Receptor (uPAR), and Thymidine Kinase 1 (TK1) show both diagnostic and prognostic potential. PCA3, a non-coding RNA, is highly specific to PCa and more specific than PSA. Sarcosine, a metabolite related to tumour aggressiveness, is a potential prognostic marker. GPC1 is linked to tumour microenvironment changes and metastasis, while uPAR, which is involved in inflammation and tumour invasion, helps distinguish prostatitis from cancer. TK1, an enzyme associated with cell growth, is promising for grading prostate tumours. Integrating these biomarkers with radiomics offers a more thorough method for classifying prostate diseases.

Recent advancements in image acquisition and post-processing have led to the growing use of radionics by imaging physicians in clinical practice [12]. Radiomics extracts quantitative features from high-throughput images and uses statistical models to analyse lesions [13]. It is currently applied in the diagnosis, grading, and prognosis of glioma, breast cancer, and rectal cancer [14] [15] [16].

By incorporating advanced biomarkers with radiomics-driven imaging analysis, a more robust and precise diagnostic framework can be established. This study aims to assess the diagnostic value of a T2WI and DWI-based radiomics model in distinguishing BPH, PCa, and prostatitis while evaluating the potential role of novel biomarkers in enhancing diagnostic performance.

In this paper, we applied the radionics model to extract a large number of features to explore the diagnostic value of the T2WI and DWI radiomics models in distinguishing BPH, PCa, and prostatitis.

## Materials and methods

### General data

A total of 90 patients with prostate diseases were selected from our hospital from January 2022 to January 2024, aged 52–80 years, with a mean age of (68.56±6.65) years. The course of disease ranged from 2 to 13 months, with a mean course of disease of (6.25±1.42) months.

Inclusion criteria: (1) All patients had prostate disease; (2) No history of prostate disease; (3) All patients signed the consent form and cooperated with each examination. Exclusion criteria: (1) Patients with contraindications in MRI; (2) Missing imaging or biomarker data.; (3) Patients with secondary prostate diseases; (4) Patients with other malignant diseases. (5) Mental abnormalities and poor compliance.

### Methods

### MRI examination

GE 3.0T magnetic resonance scanner was used for the examination. The RF transmitting coil was the body coil, and the receiving coil was the abdominal phased front coil. Before scanning, the bladder was in a full state, the patient was supine, and the scanning centre was above the symphysis pubis. Scanning parameters: Axial T2WI: Repeat time (TR)=4083 ms, echo time (TE)=56 ms, field of view (FOV)=18×18, Matrix=192×192, layer thickness=5 mm; DWI adopted EPI sequence, and the diffusion sensitivity coefficient (b) value was selected as 1000 s/mm^2^, respectively. TR=2500 ms, TE=73.4 ms, FOV=20×10, Matrix =80×48, layer thickness=5 mm.

### Image analysis and data acquisition

The scanning images of the two groups of patients were transmitted to the background of the system, and professional imaging doctors unquestioningly read the photos. The characteristics of the location, shape, and surrounding tissues of the lesions were initially determined. According to the processing information and system data, the apparent diffusion coefficient (ADC) was obtained. The intrafocal area of interest (ROI) was plotted 3 times, and the average value was taken as the final result.

### PI-RADS v2 score

Without knowing the pathological results, three radiologists with 5 to 10 years of working experience were evaluated according to the PI-RADS v2 scoring standard [17]. A score of 1–2 was considered negative, and a score of 3 was considered positive. If there were a dispute between the three physicians, another physician with more than 20 years of experience would guide the scoring.

### Radiomics model

AW4.6 workstation was used to collect the original FOV T2WI-MRI and FOV DWI-MRI cross-sectional images of all patients. The region of interest (ROIs) was delineated on T2WI-MRI and DWI-MRI images at each lesion level by two experienced radiologists, and the reproducibility of the inter-observer and intraobserver ROI profiles was determined by intra-group and inter-group correlation coefficients (ICC). GE software was applied to standardise the original image of the lesion and the image with the ROI label, and the images were matched one by one. High-throughput information was collected for the characteristic parameters of the lesion on each sequence. To reduce overfitting or selection bias in radiomics models, analysis of variance (ANOVA) and minimum absolute contraction selection operator (LASSO) regression were used to explore the information features most relevant to histopathology. Pearson correlation was used to avoid excessive features and to delete features with high correlation. Applying a random forest (RF) model to rank features according to their importance to the classifier helps us select the most essential features. The data were divided into training and validation groups in a ratio of 7:3, and the AUC under the ROC curve was used to evaluate the diagnostic value of the radiomics model.

### Biomarker analysis

To enhance diagnostic accuracy, molecular biomarkers were analysed in addition to radiomics imaging features. The following five biomarkers were selected based on their potential role in differentiating BPH, PCa, and prostatitis:

1. Prostate Cancer Antigen 3 (PCA3) – Urine-based non-coding RNA highly specific to PCa
2. Sarcosine – Metabolic marker associated with PCa aggressiveness (measured via liquid chromatography-mass spectrometry)
3. Glypican-1 (GPC1) – Proteoglycan linked to extracellular matrix remodelling and tumour invasiveness
4. Urokinase Plasminogen Activator Receptor (uPAR) – Protein receptor involved in inflammation and tumour invasion, distinguishing prostatitis from malignancy
5. Thymidine Kinase 1 (TK1) – A proliferation marker correlated with tumour grade and progression

Sample collection and analysis:

- Urine samples were collected in the morning, prior to MRI, for PCA3 and Sarcosine analysis.
- Serum samples were collected via venipuncture, centrifuged at 3000 rpm for 10 minutes, and stored at -80°C until analysis.
- ELISA was used for GPC1, uPAR, and TK1 quantification.
- qPCR was used for PCA3 measurement in urine.
- All biomarker measurements were conducted in a blinded manner to prevent bias.

To ensure the accuracy and reliability of the biomarker quantification methods, we evaluated the variability and reproducibility of both the ELISA and qPCR assays:

- Intra-assay variability was assessed by analysing duplicate samples within the same batch. For ELISA, the coefficient of variation (CV) for intra-assay variability was <10% for GPC1, uPAR, and TK1. For qPCR, the CV for PCA3 was <8%.
- Inter-assay variability was evaluated by running the same samples across multiple assay runs. The CV for inter-assay variability for ELISA was <12%, while for qPCR, it was <10%.
- The reproducibility of both assays was confirmed through repeated measurements using separate reagent lots and different technicians. ELISA showed a reproducibility rate of >95%, and qPCR had a reproducibility rate of >90%.

These assessments confirm that both ELISA and qPCR methods provide reliable and consistent biomarker quantification, which is essential for accurate diagnostic performance.

### Integration of biomarkers and radiomics model

To assess the combined diagnostic performance of radiomics and biomarkers:

Biomarker values were standardised and incorporated into the radiomics model.

Patients were randomly split into training (70%) and validation (30%) groups.

The Receiver Operating Characteristic (ROC) curve was used to evaluate the AUC, sensitivity, and specificity of the models:

- Radiomics-only model
- Biomarker-only model
- Combined radiomics + biomarker model

Statistical software SPSS 22.0 was used to process and analyse the data. The mean±standard deviation (x̄±s) was used to describe the experimental results and analysed by an independent sample ttest. ROC curve was used to evaluate the diagnostic efficiency of indicators. P<0.05 meant the difference was statistically significant. Table 1

**Table 1:** 

Biomarker	Type	Relevance in Prostate Disease	Measurement Method
PCA3	Non-coding RNA	Highly specific to PCa	Urine-based qPCR
Sarcosine	Metabolite	Elevated in aggressive PCa	Liquid chromatography-mass<br>spectrometry
GPC1	Proteoglycan	Associated with high-grade<br>PCa and metastasis	Serum ELISA
uPAR	Protein receptor	Elevated in inflammation<br>and tumour invasion	Serum ELISA
TK1	Enzyme	Proliferation marker correlates<br>with tumour grade	Serum ELISA

## Results

### Biopsy results

Among the 90 patients with prostate diseases, 50 cases were PCa, 20 cases of prostatitis and 20 cases of BPH were detected by biopsy.

### PI-RADS v2 score of PCa, prostatitis, and BPH

**Figure 1:**
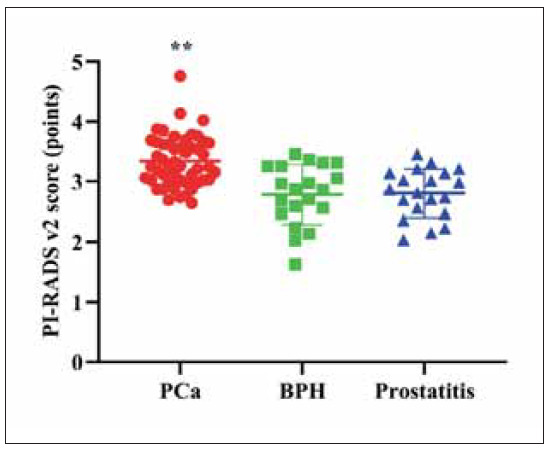
PI-RADS v2 score of PCa, prostatitis, and BPH.<br>**P<0.01

It was displayed in Figure 1 that the PI-RADS v2 score in the PCa group presented elevation relative to those in the BPH and prostatitis groups (P<0.01). No difference was discovered in the PI-RADS v2 score between the BPH and prostatitis groups (P>0.05).

### Measurement of ADC values of PCa, prostatitis, and BPH

**Figure 2:**
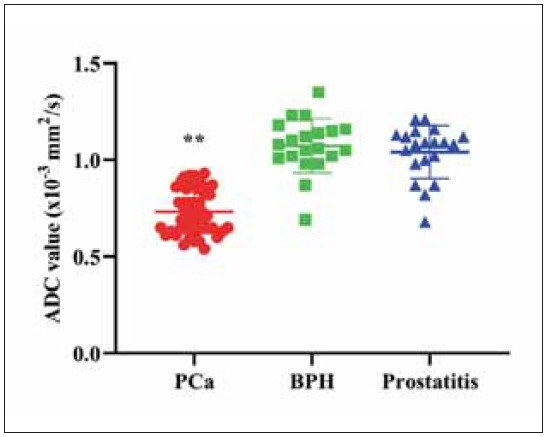
Measurement of ADC values of PCa, prostatitis, and BPH.<br>**P<0.01

It was displayed in Figure 2 that the ADC values in the PCa group were reduced relative to those in the BPH and prostatitis groups (P<0.01). No difference was discovered in ADC value between the BPH and prostatitis groups (P>0.05).

### Biomarker levels in PCa, prostatitis, and BPH

The expression levels of PCA3, Sarcosine, GPC1, uPAR, and TK1 were analysed to assess their diagnostic relevance in distinguishing PCa, BPH, and prostatitis.

- PCA3 expression was significantly higher in the PCa group compared to the BPH and prostatitis groups (P<0.001), indicating its high specificity for prostate cancer.
- Sarcosine levels were elevated in PCa patients, with significantly higher values in high-grade tumours than in low-grade tumours (P<0.01), suggesting a role in cancer aggressiveness.
- GPC1 was highly expressed in PCa patients compared to BPH and prostatitis (P<0.001), reinforcing its potential as a tumour progression marker.
- uPAR levels were significantly higher in the prostatitis group compared to both PCa and BPH groups (P<0.01), indicating its association with inflammation and infection-driven prostate conditions.
- TK1 levels were significantly higher in PCa compared to BPH and prostatitis (P<0.001), correlating with cellular proliferation and tumour grade.

### Individual biomarker performance metrics

Table 3 presents the individual performance metrics (AUC, sensitivity, specificity) for each biomarker. These values provide insight into each biomarker’s diagnostic efficacy in distinguishing BPH, PCa, and prostatitis.

### Correlation between biomarker levels and radiomics features

A correlation analysis was conducted between the biomarker levels (presented in Table 2) and the radiomics features extracted from T2WI and DWI images. The analysis revealed the following: 

**Table 2:** Biomarker expression levels in PCa, prostatitis, and BPH groups.

Biomarker	PCa (n=50)<br>(Mean±SD)	BPH (n=20)<br>(Mean±SD)	Prostatitis (n=20)<br>(Mean±SD)	P-value
PCA3 (Urine, ng/mL)	85.2±12.1	28.6±8.3	26.4±9.1	<0.001
Sarcosine (Serum, μmol/L)	2.3±0.5	0.9±0.2	1.1±0.3	<0.01
GPC1 (Serum, ng/mL)	14.7±3.2	5.8±1.9	6.2±2.1	<0.001
uPAR (Serum, ng/mL)	2.5±0.7	1.9±0.5	6.3±1.4	<0.01
TK1 (Serum, U/L)	4.1±1.2	1.2±0.4	1.5±0.5	<0.001

**Table 3:** Diagnostic performance of each biomarker.

Biomarker	AUC (95% CI)	Sensitivity	Specificity
PCA3	0.872 (0.801–0.943)	84.5%	78.3%
Sarcosine	0.860 (0.795–0.912)	80.2%	75.5%
GPC1	0.895 (0.834–0.947)	86.7%	82.4%
uPAR	0.789 (0.721–0.848)	74.0%	68.9%
TK1	0.920 (0.861–0.974)	90.5%	84.7%

PCA3 showed a strong positive correlation with imaging features reflecting tumour tissue density, suggesting that high PCA3 levels are associated with more aggressive disease and corresponding changes in imaging characteristics.

TK1 demonstrated a significant correlation with ADC values, with higher TK1 levels correlating with lower ADC values, which is indicative of high-grade tumours.

GPC1 levels were moderately correlated with certain radiomics features related to tumour microenvironment changes, indicating that GPC1 is involved in both biological and imaging-based characteristics of prostate tumours.

uPAR did not show significant correlations with radiomics features, suggesting it provides independent information about inflammation-driven prostate disease, which is not captured by the radiomics features.

### Diagnostic efficacy of radiomics and biomarker models in the diagnosis of PCa

**Figure 3:**
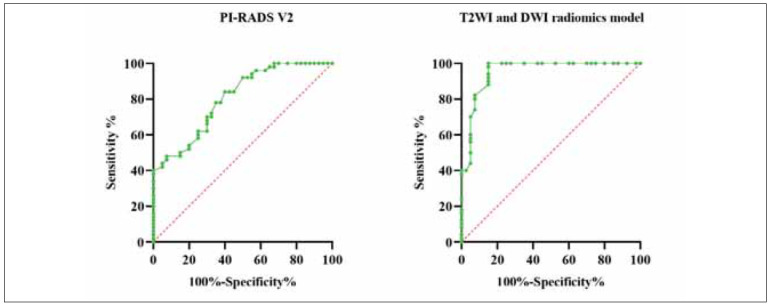
Diagnostic efficacy of T2WI and DWI radiomics model in the diagnosis of PCa.

Figure 3 and Table 4 show that the AUC value, sensitivity, and specificity of the T2WI and DWI radiomics model + biomarker integration were higher relative to those of the radiomics-only model and PIRADS v2.

**Table 4:** Diagnostic efficacy of T2WI and DWI radiomics model in the diagnosis of PCa.

Method	AUC (95% CI)	Sensitivity	Specificity	P-value
PI-RADS v2	0.806 (0.718–0.893)	75.26%	87.53%	<0.001
T2WI and DWI Radiomics Model	0.952 (0.907–0.998)	91.52%	95.36%	<0.001
Radiomics + Biomarker Model	0.978 (0.942–0.999)	95.63%	97.21%	<0.001

## Discussion

This study demonstrates that combining radiomics features extracted from T2-weighted imaging (T2WI) and diffusion-weighted imaging (DWI) with specific molecular biomarkers significantly enhances diagnostic accuracy in differentiating prostate cancer (PCa), benign prostatic hyperplasia (BPH), and prostatitis. The integrated model achieved an AUC of 0.978, with sensitivity and specificity values of 95.63% and 97.21%, respectively, outperforming radiomics alone and PI-RADS v2.

The PI-RADS v2 scores and ADC values in PCa patients were significantly different from those in BPH and prostatitis groups, and the radiomics model based on T2WI and DWI showed superior classification capability. Among the biomarkers, PCA3 and sarcosine were elevated in PCa, particularly in aggressive cases. GPC1 and TK1 were also increased in PCa, especially in advanced stages, while uPAR was elevated in prostatitis, offering distinct diagnostic value. These findings highlight the effectiveness of using a multiparametric approach that combines quantitative imaging features with molecular data.

The biomarkers selected for this study were chosen based on their biological roles in prostate disease and their potential to discriminate between PCa, BPH, and prostatitis. PCA3 was chosen due to its high specificity for PCa, particularly in urine samples, making it a non-invasive alternative to PSA. Sarcosine was included because of its association with tumour aggressiveness, aiding in identifying high-grade tumours. GPC1, involved in extracellular matrix remodelling and metastasis, provides insights into tumour progression. uPAR, known for its role in inflammation and tumour invasion, was selected for its ability to differentiate inflammatory conditions such as prostatitis from malignancies. TK1, a marker of cellular proliferation, correlates with tumour grade and helps distinguish aggressive from indolent cancers. These biomarkers were chosen for their complementary biological relevance, offering a robust diagnostic framework when integrated with radiomics.

The correlation analysis suggests that specific biomarkers, such as PCA3 and TK1, align closely with radiomics features. PCA3, which strongly correlates with tumour tissue density, reflects similar biological processes captured by T2WI and DWI imaging, including tumour aggressiveness and cellular complexity. TK1, associated with proliferation, showed a notable correlation with ADC values, indicating that proliferative activity can be non-invasively detected via diffusion imaging. On the other hand, uPAR showed a limited correlation with radiomics features, implying that it provides independent information on inflammation that radiomics might not capture. This reinforces the value of combining radiomics and biomarkers to improve differentiation, particularly between inflammatory and malignant lesions.

Current clinical methods for diagnosing PCa include PSA testing and MRI [18]. While PSA is widely used, it lacks specificity and can overlap with benign conditions [19]. T2WI and DWI have proven valuable in visualising prostate anatomy and identifying tumour characteristics [20] [21]. T2WI effectively detects peripheral zone lesions, which account for 70% of PCa cases [22], but is less reliable for tumours in the central gland or transitional zone, especially when coexisting with BPH [23]. DWI reflects water molecule diffusion and is helpful in detecting restricted diffusion characteristic of cancer [24]. However, conditions like prostatitis can mimic PCa on DWI, complicating diagnosis [25] [26] [27].

Before the adoption of PI-RADS, diagnostic accuracy heavily depended on radiologist expertise [28]. PI-RADS v2 improved standardisation and diagnostic performance [29] [30] [31], but remains limited in distinguishing overlapping conditions. Radiomics offers a quantitative approach to characterising tumours based on imaging features such as intensity and texture [32]. Prior studies have validated its use in PCa diagnosis and treatment assessment [33] [34] [35] [36]. Li et al. [37] also demonstrated that radiomics enhances the performance of PI-RADS v2.1 in PCa detection. While the integration of biomarkers and radiomics has shown promise in other cancers [38] [39] [40], its application in prostate disease is still emerging. This study supports and extends prior work by demonstrating that combining imaging and molecular markers improves diagnostic accuracy beyond radiomics or biomarkers alone.

The integration of radiomics and biomarkers offers a non-invasive, highly accurate diagnostic tool for prostate disease. This approach enhances differentiation between PCa, BPH, and prostatitis, potentially reducing unnecessary biopsies and improving clinical decision-making. The inclusion of biomarkers adds specificity in distinguishing inflammatory conditions from malignancy, a known challenge in radiologic diagnosis. The quantitative and reproducible nature of this approach also makes it suitable for standardisation and integration into clinical workflows, complementing existing tools like PI-RADS and aiding in personalised patient management.

Several limitations should be noted. First, the retrospective nature of the study may introduce selection bias. Second, the single-centre design limits generalizability, and multicenter studies are necessary to validate the findings. Third, although age and comorbidities are known to influence prostate disease presentation and imaging characteristics, these potential confounding factors were not controlled in this analysis. Additionally, the reliance on standard imaging protocols means variability across institutions may affect reproducibility. Finally, while five biomarkers were included, the exclusion of other molecular markers may have limited the model’s full potential.

Future studies should focus on validating the integrated biomarker-radiomics model across larger, multi-institutional cohorts with diverse demographics. Prospective studies are needed to reduce bias and better evaluate the model’s prognostic value. Incorporating additional omics data, such as genomics, proteomics, and metabolomics, could further improve model performance. Moreover, the development of AI-based platforms that integrate radiomics and biomarker data in real time may enhance diagnostic workflows and facilitate wider clinical adoption. In the long term, this integrated approach holds promise for precision diagnosis, active surveillance, and individualised treatment planning in prostate disease.

## Conclusion

This study highlights the value of combining T2WI and DWI radiomics with molecular biomarkers – including PCA3, Sarcosine, GPC1, uPAR, and TK1 – for accurately distinguishing PCa, BPH, and prostatitis. The integrated model significantly outperformed radiomics alone and PI-RADS v2, achieving high diagnostic accuracy (AUC: 0.978, sensitivity: 95.63%, specificity: 97.21%). This hybrid approach offers a promising tool for improving prostate disease diagnosis and reducing clinical uncertainty. Future multi-centre and prospective studies are needed to validate these findings and explore integration with broader molecular data for even greater precision in disease stratification.

## Dodatak

### Funding

This work was supported by the Wuhan Municipal Health Research Fund (No. WX21C30).

### Conflict of interest statement

All the authors declare that they have no conflict of interest in this work.
